# An Ecotoxicological Evaluation of Four Fungal Metabolites with Potential Application as Biocides for the Conservation of Cultural Heritage

**DOI:** 10.3390/toxins14060407

**Published:** 2022-06-14

**Authors:** Mariagioia Petraretti, Antonietta Siciliano, Federica Carraturo, Alessio Cimmino, Antonino De Natale, Marco Guida, Antonino Pollio, Antonio Evidente, Marco Masi

**Affiliations:** 1 Department of Biology, University of Naples Federico II, Complesso Universitario Monte Sant’Angelo, Via Cintia 4, 80126 Naples, Italy; antonietta.siciliano@unina.it (A.S.); federica.carraturo@unina.it (F.C.); denatale@unina.it (A.D.N.); marco.guida@unina.it (M.G.); antonino.pollio@unina.it (A.P.); 2 Department of Chemical Sciences, University of Naples Federico II, Complesso Universitario Monte Sant’Angelo, Via Cintia 4, 80126 Naples, Italy; alessio.cimmino@unina.it (A.C.); evidente@unina.it (A.E.)

**Keywords:** cultural heritage, biodeterioration, natural compounds, antifungal, fungi, cavoxin, *epi*-epoformin, sphaeropsidone, seiridin

## Abstract

Biocides based on chemical synthetic compounds have been commonly used to counteract damages caused by microorganisms on stone cultural heritage. However, in the last few years, the use of commercial and traditional biocides has been banned and/or limited due to their dangerous profile for the environment, as well as human and animal health. Natural products could be used as suitable alternatives for cultural heritage purposes, as they have low toxicity and stability compared with synthetic pesticides. Even if most of the investigated solutions have already shown promising results, their efficiency, ecotoxicological, and chemical features are poorly investigated. In this manuscript, we aimed to evaluate the ecotoxicological profile of four fungal metabolites—namely, cavoxin, *epi*-epoformin, seiridin, and sphaeropsidone—with potential antimicrobial properties for monumental artworks. A battery of ecotoxicological tests using *Aliivibrio fischeri* (bacterium), *Raphidocelis subcapitata* (alga), *Raphanus sativus* L. (macrophyte), *Daphnia magna* (crustacean), and *Caenorhabditis elegans* (nematode) revealed a relative lower toxicity of these compounds, especially when compared with Preventol^®^ and Rocima^®^, commercial biocides mainly used for the conservation of cultural heritage.

## 1. Introduction

All mineral building materials, including cultural heritage objectives, are subjected to microbial colonisation, which can determine their long-term biodeterioration [1,2]. This phenomenon consists of aesthetic damage, together with the loss of structural integrity and alteration of physical and chemical properties of many types of materials [3,4]. Controlling the biodeterioration of cultural heritage is becoming a major challenge, due to its heterogeneous nature dependent on biodeteriogen types and their degree of extension, as well as the artefact conservation status and environmental factors [5]. The mitigation strategies so far employed cannot completely solve the problem of biodeterioration on building materials. Among these, the most used solution to control undesirable microorganisms involved in the biodeterioration process is the application of synthetic biocides [6].

However, the European Community directive [7] has increasingly deterred the use of traditional chemical biocides, due to risks for human health and the environment, although they differ in their toxicological profile, as indicated in EU reference databases (https://echa.europa.eu/home, accessed on 18 May 2022). Indeed, the marketed broad-spectrum and harsh biocides cause human health issues for customers, especially when used without basic personal protections [8,9,10]. Moreover, studies have been demonstrating that biocides applied on cultural heritage surfaces are frequently persistent in the environment and poorly biodegradable, leading to long-term soil and water pollution [11,12]. In addition, the degradation products issued from these biocides and the possible interference on the building materials remain poorly understood and should be considered. Extensive research is now ongoing on the use of natural products as a tool to halt or reduce biodeterioration, replacing traditional chemical biocides [13,14,15]. These compounds are hypothesised to be less toxic than synthetic ones and appear promising to fight biodeterioration. In fact, they possess a wide diversity of action mechanisms (quorum sensing inhibition, modification of cell permeability, or energy metabolism), and are able to overcome the resistance of some microorganisms to chemical biocides [16]. The current state of the art shows some works based on the application of natural biocides (mainly essential oils and plant-derived compounds) to reduce the biodeterioration of cultural heritage [17,18].

**Figure 1:**
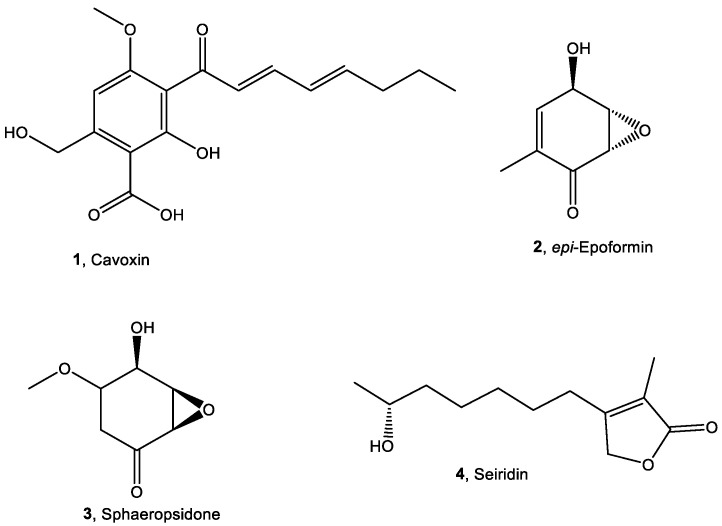
Chemical structures of compounds **1**–**4**.

Recently, the natural metabolites cyclopaldic acid, cavoxin (**1**, Figure 1), and *epi*-epoformin (**2**, Figure 1) were evaluated as potential antifungal compounds against *Aspergillus niger*, *Alternaria alternata,* and *Fusarium oxysporum*, three of the main fungi retrieved on cultural heritage in Campania, Italy [19,20].

Cavoxin and *epi*-epoformin showed antifungal activity against *A. niger* and *F. oxysporum* on infected stones of the Neapolitan yellow tuff (NYT), a volcanic lithotype widely diffused in the archaeological sites of Campania, Italy [19,20]. However, their eco-friendly compatibility is one of the most important prerequisites for their application, as well as their possible detrimental effects on different lithic materials. Despite the promising results, the knowledge about the non-target effects of these natural metabolites is unknown. Their use could reach the environmental concentrations through the release into water and sediments, which will cause biological toxic effects on a variety of organisms. After entering the environment, biocides could enter the biogeochemical cycle through a different route and could be absorbed directly by organisms and amplified through the food chain. Therefore, the biological toxicity of the biocides requires careful investigation. The Biocidal Product Directive of the European Union (BPD-98/EC) covers and rules the use of 23 chemical product groups. The aim of this directive is that all products used for biocidal purposes should be within the scope of the BPD and treated equally [21]. Thus, eco-sustainability assessment through standard protocol should also be a part of the selection of natural biocides. To this end, five living organisms—the bacterium *Aliivibrio fischeri*, the algae *Raphidocelis subcapitata*, the crustacean *Daphnia magna*, the nematode *Caenorhabditis elegans*, and macrophyte *Raphanus sativus*—were used as bioindicators for ecotoxicology evaluation of these fungal metabolites. Furthermore, other two promising antimicrobial metabolites—namely, sphaeropsidone (**3**, Figure 1) [22] and seiridin (**4**, Figure 1) [23]—were also evaluated.

## 2. Results and Discussion

The fungal metabolites used in this study (**1**–**4**, Figure 1), belonging to different classes of natural compounds, were isolated from the culture filtrates of four phytopathogenic fungi [24,25]. In particular, the 2,3,4,6-tetrasubstituted benzoic acid cavoxin (**1**) was produced in vitro by the phytopathogenic fungus *Phoma cava* isolated from an infected chestnut tree [25]. The two cyclohexenes oxide *epi*-epoformin (**2**) and sphaeropsidone (**3**) were isolated from the pathogens of oak plants *Diplodia quercivora* [26] and *Diplodia cupressi* [22,27], respectively. The butenolide seiridin (**4**) was produced in vitro by *Seiridium cardinale*, a fungus responsible for cankers of Italian cypress (*Cupressus sempervirens* L.) [23]. Their spectroscopic (^1^H NMR and ESI MS) and optical data were in agreement with those previously reported in the literature [22,23,25,26,27,28].

Compounds **1**–**4** showed antimicrobial activities against different microorganisms. In particular, cavoxin (**1**), besides showing toxicity against *A. niger* and *F. oxysporum* [20], demonstrated antifungal activity towards pea powdery mildew incited by *Erysiphe pisi* [29] and plant pathogenic fungi such as *Colletotrichum acutatum* and *Colletotrichum fragariae* [30]. *epi*-Epoformin was active against *A. niger* and *F. oxysporum* [20] and inhibited the germination and penetration of rusts *Puccinia* sp. and *Uromyces* sp. [31,32]. Sphaeropsidone (**3**) showed antifungal activity against *Seiridium* spp. (namely, *S. cardinale*, *S. cupressi* and *S. unicorne*), *Botrytis cinerea, Phomopsis amygdali* [22], and five fungal species belonging to the genus *Phytophthora* [27], while seiridin (**4**) showed antibacterial activity against *Bacillus megaterium* and *Pseudomonas fluorescens* [33].

Recently, these compounds have been tested to evaluate their toxicity against zebrafish embryos and mammalian cells, together with other natural products with promising biological activities. At a concentration of 5, 25, or 50 mM, they did not exhibit any adverse effects on hatching rate and embryo viability, either during the embryonic or the larval stages of zebrafish [34]. However, the ecotoxicological profiles of compounds **1**–**4**, as well as their chemical stability in the medium used for these tests, were never investigated.

Thus, the stability of compounds **1**–**4** in ISO culture medium after 72 h (corresponding to the longest time used for the algal inhibition test) was evaluated by qualitative and quantitative analyses. For the qualitative analysis, the solutions of ISO medium containing the four metabolites were extracted three times with EtOAc, and the corresponding organic extracts were analysed via TLC in comparison with standard samples of compounds **1**–**4**. Among the metabolites under study, only cavoxin (**1**) was not detected in the corresponding organic extract whose chromatographic profile showed the presence of other compounds (probably degradation products). The extract of the ISO medium without the compounds did not show any spots in correspondence to those of compounds **1**–**4**.

To confirm the stability of the other compounds (**2**–**4**), and the result obtained with cavoxin, a quantitative analysis was carried out using HPLC. Standard samples of compounds **1**–**4** were used to obtain HPLC calibration curves (Table 1) for their quantitative determination in ISO medium solutions after 72 h.

**Figure 2:**
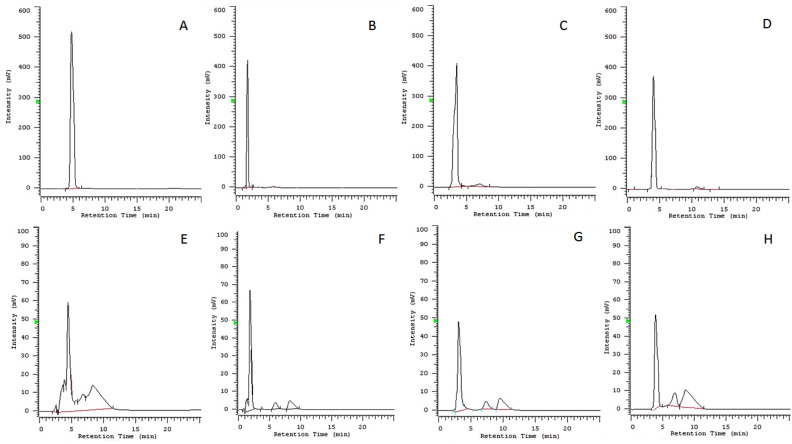
Chromatographic profiles: (**A**) standard sample of cavoxin (**1**), 1 μg/mL at 286 nm; (**B**) standard sample of *epi*-epoformin (**2**), 1 μg/mL at 237 nm; (**C**) standard sample of sphaeropsidone (**3**), 1 μg/mL at 257 nm; (**D**) standard sample of seiridin (**4**), 1 μg/mL at 215 nm; (**E**) culture medium (ISO medium 2012) 72 h after the addition of cavoxin (**1**), 0.1 μg/mL at 286 nm; (**F**) culture medium (ISO medium 2012) 72 h after the addition of *epi*-epoformin (**2**), 0.1 μg/mL at 237 nm; (**G**) culture medium (ISO medium 2012) 72 h after the addition of sphaeropsidone (**3**), 0.1 μg/mL at 257 nm; (**H**) culture medium (ISO medium 2012) 72 h after the addition of seiridin (**4**), 0.1 μg/mL at 215 nm.

The retention times were highly reproducible, varying less than 0.500 min. Linear regression curves (absolute amount against chromatographic peak area) were obtained for **1**–**4** based on weighted values calculated for seven concentrations of the standards. The quantitative determination of the metabolites was calculated by interpolating the mean area of the chromatographic peak using the equation from the calibration curve. The chromatographic profiles of standard samples of compounds **1**–**4** and those of the solutions obtained adding to ISO medium the same compounds after 72 h are reported in Figure 2.

The peak of cavoxin was absent in the corresponding chromatographic profile (Figure 2E), confirming the result obtained with the qualitative TLC analysis. Instead, the peaks of compounds **2**–**4** in the HPLC profile (Figure 2F–H) were almost coincident with the retention times of standards (Figure 2B–D), and the percent concentration of compounds present in the culture medium after 72 h is reported in Table 2. Furthermore, when ISO medium without the compounds was analysed at 215 nm, no significant peaks were observed in correspondence with the retention times of compounds **1**–**4** (Supplementary Materials, Figure S1). The results showed that compounds **2**–**4** were still present in high concentrations (≥90%) in ISO medium solution at 72 h, confirming their stability in these conditions. The stability of **2**–**4** solutions ensured that test results in this research were not altered by this variable during the exposure period.

In Figures S2–S5, the results of the effect mean percentage of *R. subcapitata*, *A. fischeri*, *D. magna*, and *C. elegans* are reported for **1**–**4**; the equations allowing the determination of EC_50_, EC_20_, and EC_5_ are also included in Figures S2–S5.

Algae are primary producers and the basis of the food chain in aquatic scenarios and ecotoxicological effects range between inhibition or stimulation of their growth. In Table 2, EC_50_, EC_20_, and EC_5_ values are reported for *R. subcapitata* exposed to different concentrations of compounds **1**–**4**. These results showed that all the compounds (**1**–**4**) displayed the following increasing order of toxicity according to the estimates obtained: for EC_50_, Ec_20_, and EC_5_, **4** < **1** < **3** < **2**. Therefore, the algal toxicity for **2** and **3** was higher than the toxicities of **1** and **4**, which presented low or no toxicity, respectively.

*A. fischeri* bacteria are decomposers, and their luminescence decreases when their metabolism is damaged by contaminants. Table 2 shows the effective concentrations due to the variation in luminescence when the bacteria were exposed to compounds **1**–**4**.

For *A. fischeri*, EC_50_ values of 68.14 mg/L and 30.96 mg/L were obtained from the tested concentrations of **3** and **4**, respectively. The EC_50_ value of **1** was 8.57 mg/L, while the EC_50_ value of **2** resulted in 5.12 mg/L. Based on these values, **4** and **3** displayed lower toxicity than **1** and **2**; thus, the dilution of these compounds following their environmental applications will not significantly disturb bacterial metabolism in aquatic ecosystems.

The freshwater crustacean *D. magna* is widely distributed in freshwater environments, making it a representative primary consumer for conducting environmental hazard assessments.

Table 2 shows the EC_50_, EC_20_, and EC_5_ values in relation to immobility of *D. magna* after exposure to different concentrations of **1**–**4**. These results were ranked in increasing order of toxicity for EC_50_, EC_20_, and EC_5_ as follows: **4** < **1** < **3** < **2**. Apart from **1**, the estimated effective concentrations were very close to values observed for the algae *R. subcapitata*.

The nematode *C. elegans* is well-suited for ecotoxicological experiments, and data generated for these organisms exposed to **1**–**4** are shown in Table 2. EC_50_ values were very low for **1**–**3** (range between 3.12 mg/L and 9.44 mg/L), showing a higher sensitivity of this organism to the natural biocides. Additionally, in this case, **4** did not show any toxicity.

The phytotoxicity data obtained for *R. sativus* exposed to compounds **1**–**4** are not shown, because the results indicated that the response of macrophytes to the dilutions of compounds **1**–**4** was not sufficient to calculate any effective concentrations. Considering the low toxicity and the low differences in species sensitivities between macrophytes, no additional studies were necessary to evaluate toxicity in other plant species.

The experimental data from these organisms were used for the estimation of effective concentrations (EC), which serve as the basis for a comparative analysis of the sensitivity of these organisms to different compounds. For **1**, by analysing Table 2, similar tendencies were found for the *A. fischeri, D. magna*, and *C. elegans*, while microalgae showed lower sensitivity, indicating that chronic effects in the growth inhibition test were at least 4-, 18-, and 3-fold below the concentrations that caused luminescence inhibition, immobility, and mortality, respectively.

As regards the EC values of the other compounds, (**2**) and (**3**), the same range of toxicity was found on the tested organisms, suggesting similar responses of organisms at those concentrations. Results from this study highlighted that the biological response to the same biocides was strongly dependent on the sensitivity of the species. The immobility of *D. magna* was more sensitive to cavoxin (**1**) than to the algal growth of *R. subcapitata*, whereas the algal growth was more sensitive to sphaeropsidone (**3**). The mortality of *C. elegans* was the more sensitive biological response to epi-epoformin (**2**), whereas the luminescence of *A. fischeri* was the only response sensitive to seiridin (**4**). Therefore, the toxicity of these biocides seems to be species-specific, and it also depends on the diversity of chemical structure and the biological response chosen. These results were in line with a previous study on the comparative toxicity of alternative antifungal biocides [35].

Our results indicated that seiridin (**4**) should be considered the least toxic compound. According to CLP Regulation, which detects the hazardous chemicals and the risks associated with them, compound **4** was the only tested biocide that could be listed as non-toxic for the environment (EC_50_ > 100 mg/L). The others could be labelled as harmful to aquatic life (10 < EC_50_ < 100 mg/L), following UN standard labelling guidelines (e.g., UN, 2011) [36]. Cavoxin, *epi*-epoformin, and sphaeropsidone, albeit derived from natural products, should not be evaluated as ‘green’ or ‘greener’, *per se*, but be considered as having the lowest toxicity levels found, compared with commercial biocide preparations. For example, Preventol^®^, largely used to control microbial growth in monuments, is fatally toxic to many living organisms (EC_50_ < 1 mg/L) and may pose a hazard to humans, animals, as well as to the environment [37]. EC values higher than commercial biocides identify natural biocides as a category harmful to aquatic life in the acute toxicity hazard class but necessarily less toxic than a chemical assigned a category fatally toxic to many living organisms. Therefore, our data support that cavoxin (**1**), *epi*-epoformin (**2**), and sphaeropsidone (**3**) caused deleterious effects on exposed organisms to those concentrations, whilst seiridin (**4**) showed no toxic effects on the biological responses tested here at worst-case environmental concentrations.

As the first reported findings of **1**–**4** about their effective concentrations, these results could be very useful for environmental managers and the scientific community. Considering the limited number of organisms only recently tested, this study should be considered the first attempt towards a detailed investigation of environmental problems due to the application of natural biocides.

## 3. Conclusions

The adoption of effective, eco-friendly, and cheap biocides to control biodeterioration is crucial in conservation and restoration fields. The identification of chemical-stable and long-lasting natural compounds confirms the possible use of fungal metabolites in the development of conservation products capable of preventing biological colonisation in the medium/long term. The idea that compounds deriving from natural products are mandatorily biocompatible and environmentally friendly was challenged in this study, by addressing the ecotoxicity of cavoxin (**1**), *epi*-epoformin (**2**), seiridin (**3**), and sphaeropsidone (**4**) towards five standard organisms. Therefore, our data support that cavoxin (**1**), *epi*-epoformin (**2**), and sphaeropsidone (**3**) caused deleterious effects on exposed organisms to those concentrations, except if referred to macrophyte *R. sativus*. By contrast, seiridin (**4**) showed no toxic effects on the biological responses tested at worst-case environmental concentrations. However, comparing the results obtained with compounds **1**–**4** with those obtained using the commercial biocides Preventol^®^ and Rocima^®^, relatively lower levels of toxicity were observed in all the organisms.

Thus, the obtained results must be considered the first attempt in an innovative line of research, which prompt us to conduct other investigations. In this perspective, further assays need to be performed to evaluate the inhibition activity and eco-compatibility of the identified compounds. Afterwards, experimental studies must be carried out for the purpose of evaluating the possible interaction between the biocide compounds and cultural heritage materials and to develop eco-friendly processes for their large-scale production and suitable formulations.

## 4. Materials and Methods

### 4.1. Instruments and Chemicals

Column chromatography (CC) was performed using silica gel (Kieselgel 60, 0.063–0.200 mm, Merck, Darmstadt, Germany). Thin-layer chromatography (TLC) was performed on analytical and preparative silica gel plates (Kieselgel 60, F_254_, 0.25 and 0.5 mm, respectively, Merck, Darmstadt, Germany); the spots were visualised via exposure to UV light (254 nm) and/or iodine vapours and/or by spraying first with 10% H_2_SO_4_ in MeOH and then with 5% phosphomolybdic acid in EtOH, followed by heating at 110 °C for 10 min. ^1^H NMR spectra were recorded at 500 MHz, in CDCl_3_ on a Varian spectrometer (Palo Alto, CA, USA), and the same solvents were used as internal standards. Electrospray ionisation mass spectra (ESIMS) were performed using the LC/MS TOF system AGILENT 6230B (Agilent Technologies, Milan, Italy), HPLC 1260 Infinity. A JASCO P-1010 digital polarimeter was used to measure the specific optical rotations. The HPLC system (HITACHI) (Merck, Darmstadt, Germany). Consisted of a pump (5160) and a spectrophotometric detector (5410). The high-performance liquid chromatography (HPLC) separations were performed using a Merck (Darmstadt, Germany) C_18_ reversed-phase column Lichrocart (250 × 4.6 mm i.d.; 5 μm). Sigma-Aldrich Co. (St. Louis, MO, USA) supplied all the reagents and the solvents.

### 4.2. Production, Isolation, and Identification of Selected Compounds

Cavoxin, *epi*-epoformin, sphaeropsidone, and seiridin (**1**–**4**, Figure 1) were purified from the culture filtrates of *Phoma cava*, *Diplodia quercivora*, *Seiridium cardinale*, and *Diplodia cupressi*, respectively. These fungi were isolated and grown in liquid culture as previously described [23,25,26,27].

Briefly, the culture filtrate (1 L) of *P. cava* was extracted with CHC1_3_ (4 × 500 mL), yielding 350 mg of organic extract, which was chromatographed on a Sephadex LH-20 column eluted with CHCl_3_-*iso*PrOH (9:1). The crude oily residue obtained was crystallised with EtOAc-petroleum ether, to yield cavoxin (**1**) as pale yellow needles (108 mg). Its ESIMS showed the protonated adduct [M + H]^+^ ion at *m*/*z* 321. Its ^1^H-NMR spectrum (Figure S6) was in agreement with the data reported in the literature [25].

The culture filtrate (1 L) of *D. quercivora* was acidified to pH 4 with 2 M HCl and extracted with EtOAc, yielding a brown oil residue (200 mg). This latter was purified via column chromatography (CC) on silica gel, eluted with CHCl_3_-*iso*PrOH (95:5), and then via CC on reverse phase eluted with Me_2_CO:H_2_O (7:3), yielding *epi*-epoformin (**2**, 76.1 mg) as a white solid. Its ESIMS showed the protonated adduct [M + H]^+^ ion at *m*/*z* 141. Its ^1^H-NMR spectrum (Figure S7) and specific optical rotation value ([α]^25^_D_ +244.5 (*c* 0.5 in EtOH) were in agreement with the data reported in the literature [26,28].

The culture filtrate (1 L) of *D. cupressi* was acidified to pH 4 with formic acid and extracted with EtOAc obtaining 420 mg as a brown-red oil. This organic extract was purified via CC on silica gel eluted with CHCl_3_-*iso*PrOH (95:5), affording nine groups of homogeneous fractions. The residues of the third fraction were crystallised from EtOAc-*n*-hexane (1:5), yielding sphaeropsidone (**3**, 153.3 mg) as white needles. Its ESIMS showed the protonated adduct [M + H]^+^ ion at *m*/*z* 156. Its ^1^H-NMR spectrum (Figure S8) as well as its specific optical rotation value ([α]^25^_D_ −129.5 (*c* 0.5 in MeOH) were in agreement with the data reported in the literature [22].

The culture filtrate (1 L) of *S. cardinale* was acidified at pH 4 with formic acid and extracted with *tert*-butyl-ethyl ether yielding a brown oily residue (220 mg). This was fractionated by successive steps of CC on silica gel, using CHCl_3_-*iso*PrOH (9:1) as eluent, and TLC using petroleum ether-Me_2_CO (6:4) as eluent, affording seiridin (**4**) (49.5 mg) as a pure compound. Its ESIMS showed the protonated adduct [M + H]^+^ ion at *m*/*z* 213. Its ^1^H-NMR spectrum (Figure S9), as well as its specific optical rotation value ([α]^25^_D_ −129.5 (*c* 0.5 in MeOH) were in agreement with the data reported in the literature [23].

### 4.3. Stability Studies on the Selected Compounds

#### 4.3.1. Qualitative Analysis

For this procedure, 10 mg of pure cavoxin, *epi*-epoformin, sphaeropsidone, and seiridin (**1**–**4**, Figure 1) were separately added to 100 mL of the corresponding culture medium ISO 2012 [38]. After 72 h (corresponding to the longest time used for the algal inhibition test), the metabolites were extracted from 50 mL of the culture media with EtOAc (3 × 50 mL), obtaining 4.90, 4.89, 4.80, and 4.95 mg for compounds **1**–**4**, respectively. The four extracts were analysed via TLC eluted with CHCl_3_-*iso*PrOH 95:5 (*v*/*v*) in comparison with standard samples of compounds **1**–**4**. Then, 50 mL of culture media, without any compound added, was extracted in the same conditions, obtaining 0.19 mg of organic extract.

#### 4.3.2. Quantitative Analysis

The HPLC analysis was carried out on the same solution of ISO medium containing compounds **1**–**4** after 72 h. The mobile phase used to elute the samples in isocratic mode was MeCN–H_2_O 70/30 (*v*/*v*) at a flow rate of 0.5 mL/min. Detection was performed at 286, 237, 257, and 215 nm, corresponding to the maximum UV absorption of cavoxin (**1**) [25], *epi*-epoformin (**2**) [26], sphaeropsidone (**3**) [27], and seiridin (**4**) [23], respectively. ISO medium [37] without the compounds was analysed in the same conditions. Samples were injected using a 10 μL loop and monitored for 25 min. The same conditions were used to obtain the calibrations curves for compounds **1**–**4** whose standards were accurately weighed (±0.0001 mg) and separately dissolved in MeCN in the range between 1 and 0.0001 μg/mL. Each analysis was performed in triplicate. The limit of detection (LOD) was extrapolated from the calibration graphics according to the guidelines provided by IUPAC, while the validation of the HPLC method (in terms of limit of quantitation (LOQ), intra- and inter-assay precision, and accuracy) was achieved following the rules reported in the ‘Guidance for Industry-Bioanalytical Method Validation’ of the Food and Drug Administration (FDA, USA), as previously reported [38].

### 4.4. Ecotoxicity Analysis

#### 4.4.1. Algal Growth Inhibition

The algal growth inhibition test (72 h) with *R. subcapitata,* was carried out according to ISO 8692 [37]. The algal density was determined via spectrophotometric analysis (DR5000, Hach Lange GbH, Weinheim, Germany) at 670 nm. The percentage growth inhibition (GI, %) was calculated as the difference between the growth rate of the control group and of the sample and expressed as the mean (±standard deviation). Toxicity tests were carried out in triplicate.

#### 4.4.2. Luminescence Bacteria Inhibition

The bioluminescence inhibition test (30 min) was detected with the *A. fischeri* (NRRLB-11177) supplied by MicroBioTest, Gent, Belgium, and according to ISO 11348-3 [39]. The bioluminescence was determined using a luminometer Microtox (Model 500 analyser, New Castle, DE, USA) at 490 nm. To provide the required osmotic pressure for the bacterium, the test was conducted using a saline water solution (2% sodium chloride, NaCl). Toxicity tests were performed in triplicate with a control sample, and the percentage luminescence inhibition (LI, %) was expressed as the ratio of the decrease in bacterial light production to the remaining light.

#### 4.4.3. Crustacean Immobility

The immobility test (24 h) with *D. magna* was conducted according to ISO 6341 [40]. *D. magna* samples were selected from laboratory stock cultures at the Hygiene Laboratory of the Department of Biology of the University of Naples Federico II, in ISO medium, and daily fed with microalgae *R. subcapitata.* Groups of 5 neonates (third brood, <24 h old) in 10 mL ISO medium were exposed to each compound (*n* = 4 test groups per concentration) (ISO, 2012). After exposure, any immobility or abnormal appearance was recorded using a stereomicroscope (LEICA EZ4-HD).

#### 4.4.4. Nematocidal Activity

Mortality tests (24 h) with *C. elegans* (wild-type strain N2 variant Bristol) were performed using age-synchronous L4-larval nematodes. Ten organisms were plated into 24-well tissue culture plates containing 0.5 mL of each sample. All treatments were performed in triplicate and without feeding. After exposure, the number of dead worms was determined via a stereomicroscope (LEICA EZ4-HD).

#### 4.4.5. Root Growth Inhibition

The *R. sativus* germination and root elongation toxicity tests were performed according to ISO 11269 [41]. Macrophyte seeds (*n* = 25) were selected, disinfected with 0.1% KMnO_4_ (weight percentage, *w*/*w*) for 15 min, and then flushed with double distilled water. Afterwards, the seeds were placed on filter paper Whatman n. 1, imbibed with 5 mL of testing solution in triplicate in Petri dishes. The Petri dishes were incubated in the artificial climate chamber at 25 ± 1 °C in darkness, and the number of seeds germinated and the lengths of the developing roots were measured after 72 h. Negative controls were in distilled water. Germination (%), and root elongation inhibition were combined to calculate the germination index (G, %) [42].

### 4.5. Data Analyses

EC_50_ values, as recommended in ISO guidelines, were estimated by using linear concentration–response regression and were plotted using GraphPad Prism ver. 8 (GraphPad Software, San Diego, CA, USA). In addition, EC_5_ and EC_20_ values were calculated from a concentration–response regression derived using GraphPad Prism ver. 8.

Then, the four compounds were classified by their EC_50_ values according to CLP regulation, whereby EC_50_ < 1.0 mg/L is labelled as ‘very toxic to aquatic organisms’; 1.0 > EC_50_ < 10 mg/L is ‘toxic to aquatic organisms’; 10–100 mg/L are classified as ‘harmful to aquatic organisms’; and chemicals with an EC_50_ > 100 mg/L are considered without toxicity.

## Figures and Tables

**Table 1:** Analytical characteristics of calibration curves ^a^ and quantification of compounds **1**–**4** culture medium (ISO 2012) after 72 h.

Compound	Rt (min)	R^2^	Detection Limit (μg)	Compound Detected (μg) in 10 μL	% of Compound Present in the Culture Medium after 72 h
cavoxin (**1**)	4.790	0.9998	0.0001	n.d. ^b^	0
*epi*-epoformin (**2**)	1.830	0.9997	0.0001	0.00095 ± 0.0001	95
sphaeropsidone (**3**)	3.200	0.9998	0.0001	0.00092 ± 0.0002	92
seiridin (**4**)	4.080	0.9996	0.0003	0.00090 ± 0.0003	90

^a^ Calculated in the form y = a + bx, where y is the chromatographic peak area and x is the μg of compound with a number of data points = 21; ^b^ n.d. = not detected.

**Table 2:** EC_5_, EC_20_, and EC_50_ values for cavoxin, *epi*-epoformin, seiridin, and sphaeropsidone after exposure to *R. subcapitata*, *A. fischeri*, *D. magna*, and *C. elegans* ^a^.

Organism	Compound	EC_5_	EC_20_	EC_50_
	cavoxin (**1**)	15.97 (10.11–25.13)	20.93 (13.25–32.96)	35.98 (22.77–56.67)
*Raphidocelis subcapitata*	*epi*-epoformin (**2**)	1.91 (1.28–2.99)	2.58 (1.70–4.10)	4.67 (2.98–7.70)
	sphaeropsidone (**3**)	1.78 (1.32–2.45)	3.43 (2.49–4.82)	12.78 (8.95–18.64)
	seiridin (**4**)	n.d.	n.d.	n.d.
	cavoxin (**1**)	1.42 (0.93–2.24)	2.59 (1.66–4.16)	8.57 (5.24–14.42)
*Aliivibrio fischeri*	*epi*-epoformin (**2**)	0.59 (0.33–1.15)	1.20 (0.64–2.48)	5.12 (2.50–11.58)
	sphaeropsidone (**3**)	4.12 (3.29–5.15)	10.49 (8.38–13.14)	68.14 (54.43–85.29)
	seiridin (**4**)	5.69 (2.95–11.00)	10.01 (5.18–19.35)	30.96 (16.02–59.84)
	cavoxin (**1**)	2.64 (0.22–1.37)	4.36 (0.38–2.20)	1.91 (1.11–5.66)
	*epi*-epoformin (**2**)	1.89 (1.13–3.36)	2.68 (1.57–4.84)	5.36 (3.03–10.04)
*Daphnia magna*	sphaeropsidone (**3**)	4.26 (1.20–5.06)	6.49 (1.83–12.96)	15.07 (4.26–16.34)
	seiridin (**4**)	n.d.	n.d.	n.d.
	cavoxin (**1**)	1.98 (1.08–3.91)	3.34 (1.77–6.80)	9.44 (4.73–20.52)
	*epi*-epoformin (**2**)	1.10 (0.63–2.05)	1.56 (0.88–2.97)	3.12 (1.70–6.19)
*Caenorhabditis elegans*	sphaeropsidone (**3**)	1.12 (0.58–2.54)	2.05 (1.00–5.02)	6.88 (2.97–19.69)
	seiridin (**4**)	n.d.	n.d.	n.d.

^a^ Values are in mg/L; n.d. = not determined; EC = effective concentration; average EC values are provided with ±95% confidence limit values in brackets (*n* = 3).

## Supplementary Materials

The following are available online at https://www.mdpi.com/article/10.3390/toxins14060407/s1, Figure S1: Chromatographic profile of culture medium (ISO medium 2012) 1 μg/mL at 215 nm, Figure S2: Concentration–response relationship of **1** (A), **2** (B), **3** (C), and **4** (D) exposed to *R. subcapitata*; concentrations in the *x*-axis are expressed as mg/L: GI = growth inhibition, Figure S3: Concentration–response relationship of **1** (A), **2** (B), **3** (C), and **4** (D) exposed to *A. fischeri*; concentrations in the ↓ are expressed as mg/L: LI = luminescence inhibition, Figure S4: Concentration–response relationship of **1** (A), **2** (B), **3** (C), and **4** (D) exposed to *D. magna*; concentrations in the ↓ are expressed as mg/L: I = immobility, Figure S5: Concentration–response relationship of **1** (A), **2** (B), **3** (C), and **4** (D) exposed to *C. elegans*; concentrations in the ↓ are expressed as mg/L: M = mortality, Figure S6: ^1^H NMR spectrum of cavoxin, **1** (CDCl_3_, 500 MHz), Figure S7: ^1^H NMR spectrum of *epi*-epoformin, **2** (CDCl_3_, 500 MHz), Figure S8: ^1^H NMR spectrum of sphaeropsidone, **3** (CDCl_3_, 500 MHz), Figure S9: ^1^H NMR spectrum of seiridin, **4** (CDCl_3_, 500 MHz).
