# Supplementary material for: An Ecotoxicological Evaluation of Four Fungal Metabolites with Potential Application as Biocides for the Conservation of Cultural Heritage
Source: Toxins (Basel). 2022 Jun 14;14(6):407. doi: 10.3390/toxins14060407 (PMC9227397; doi:10.3390/toxins14060407)
Supplement: Supplementary file 1 [file toxins-14-00407-s001.zip › toxins-1756339-supplementary.pdf]

Supplementary Materials: An Ecotoxicological Evaluation of Four Fungal Metabolites with Potential Application as Biocides for the Conservation of Cultural Heritage

Mariagioia Petraretti, Antonietta Siciliano, Federica Carraturo, Alessio Cimmino, Antonino De Natale, Marco Guida, Antonino Pollio, Antonio Evidente and Marco Masi

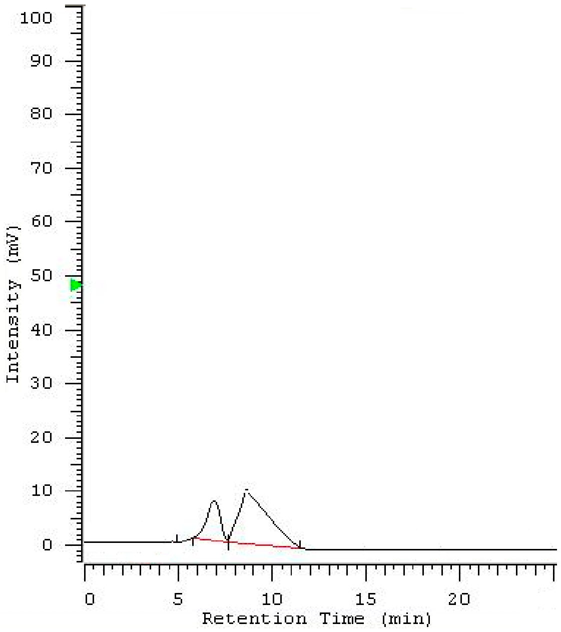

Figure S1. Chromatographic profile of culture medium (ISO medium 2012) 1 µg/mL at 215 nm.

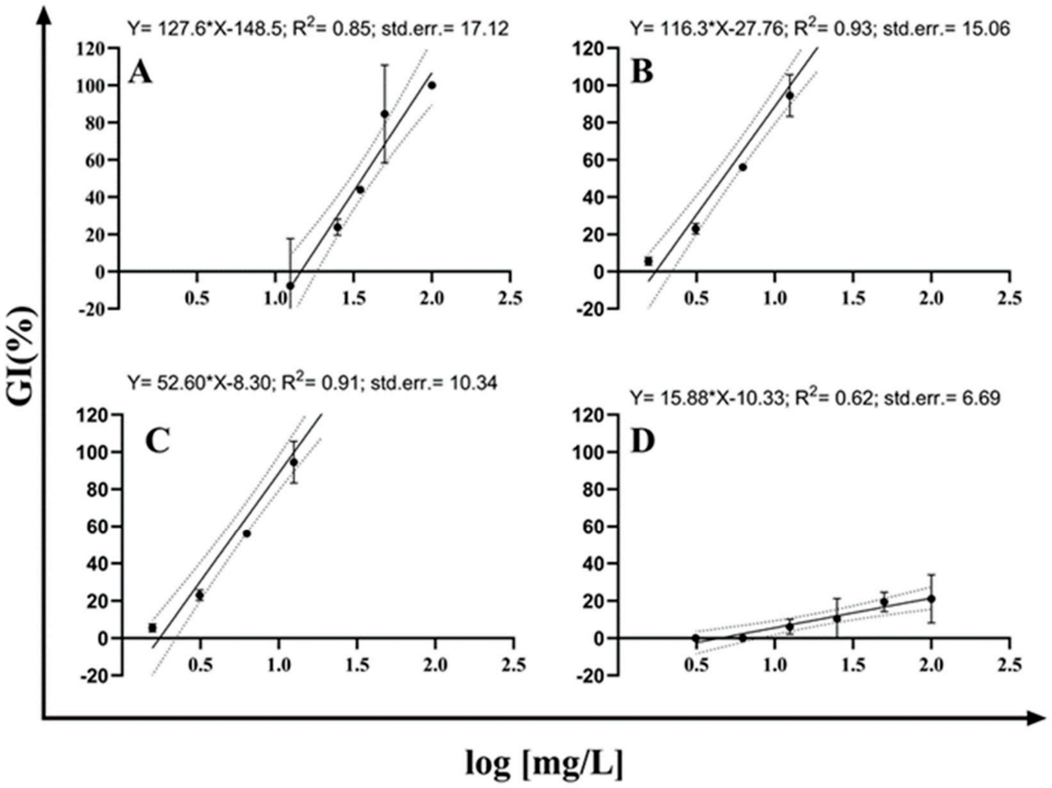

Figure S2. Concentration–response relationship of **1** (A), **2** (B), **3** (C), and **4** (D) exposed to *R. subcapitata*; concentrations in the x-axis are expressed as mg/L: GI = growth inhibition. Limits of the 95% confidence intervals are indicated by the dashed line, fitted log-linear model is indicated by the solid line.

In Figure S2, the results of the GI(%) of *R. subcapitata* are reported for **1**-**4** (Figure S2 A-D). No significant effects were detected at the first lowest exposure concentrations for **1**, **2**, and **3**, and all the concentrations for **4**. Results of *R. subcapitata* showed that the relative toxicity order was **2** > **3**> **1**> **4**. For compound **1**, biostimulation effects were detected at the first lowest exposure concentration. Algae growth impairment occurred for compounds **2** and **3** between 3.125 and 25 mg/L (nominal concentrations), and for compound **1,** between 25 and 50 mg/L. For *R. subcapitata* exposed to **1**, all concentrations provided evidence of concentration–response correlation, and their effects ranged between −8% (12.5 mg/L) and 84% (50 mg/L). For **2**, a significant difference in the concentration–response curves can be observed in Figure S2B, and the maximum detected effect was 94%. For **4**, effects ranged between 6% (12.5 mg/L) and 21% (100 mg/L) (Figure S2C), while for **3**, varied between 21% (at 3.125 mg/L) and 90% (at 50 mg/L) (Figure S2D).

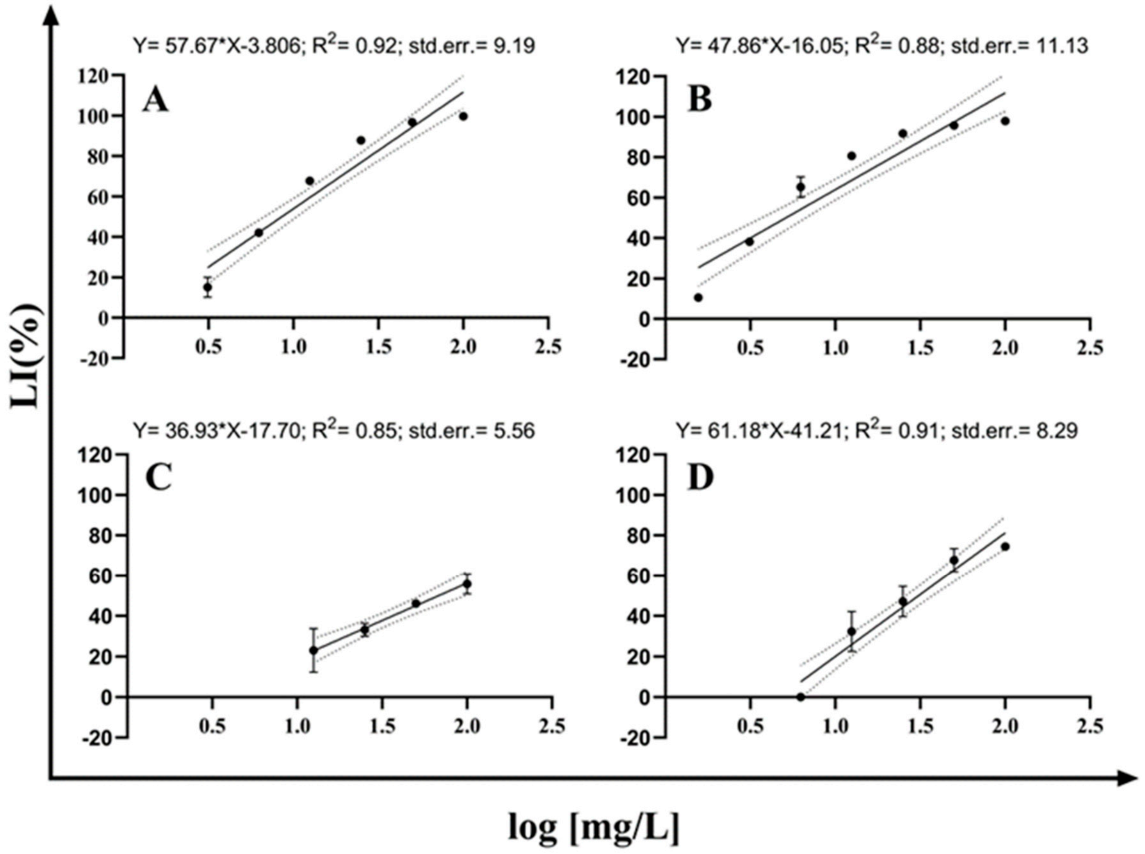

Figure S3. Concentration–response relationship of **1** (A), **2** (B), **3** (C) and **4** (D) exposed to *A. fischeri*; concentrations in the x-axis are expressed as mg/L: LI = luminescence inhibition. Limits of the 95% confidence intervals are indicated by the dashed line, fitted log-linear model is indicated by the solid line. Limits of the 95% confidence intervals are indicated by the dashed line, fitted log-linear model is indicated by the solid line.

In Figure S3, the results of the LI (%) of *A. fischeri* are reported for compounds **1**-**4** (Figure S3 A-D). As a general overview of the obtained results, LI always provided evidence of inhibitory effects at the two highest tested concentrations for the four investigated compounds. Considering the exposure of *A. fischeri* to **1** (Figure S3 A), from 12.5 mg/L to 100 mg/L concentrations, the luminescence was reduced up to 67%. At the two lowest concentrations, the inhibitory effects were considered not harmful (< 40% effect).

The luminescence of *A. fischeri* after *epi*-epoformin exposure significantly ($p < 0.05$) decreased—from 6.25 mg/L to 100 mg/L (Figure S3 B). Effects of **2** were not significantly different from those of the control at 3.12 mg/L. Among the battery of organisms selected for the test, *A. fischeri* was the only one that showed sensitivity towards this compound. The luminescence was reduced up to 75% in the considered concentration range (Figure S3 C). When **4** was tested at the highest concentrations (100 mg/L and 50 mg/L), it showed slightly adverse effects, ranging between 46% and 55%, while the two treatments at the lowest concentration had no effect (Figure S3 D).

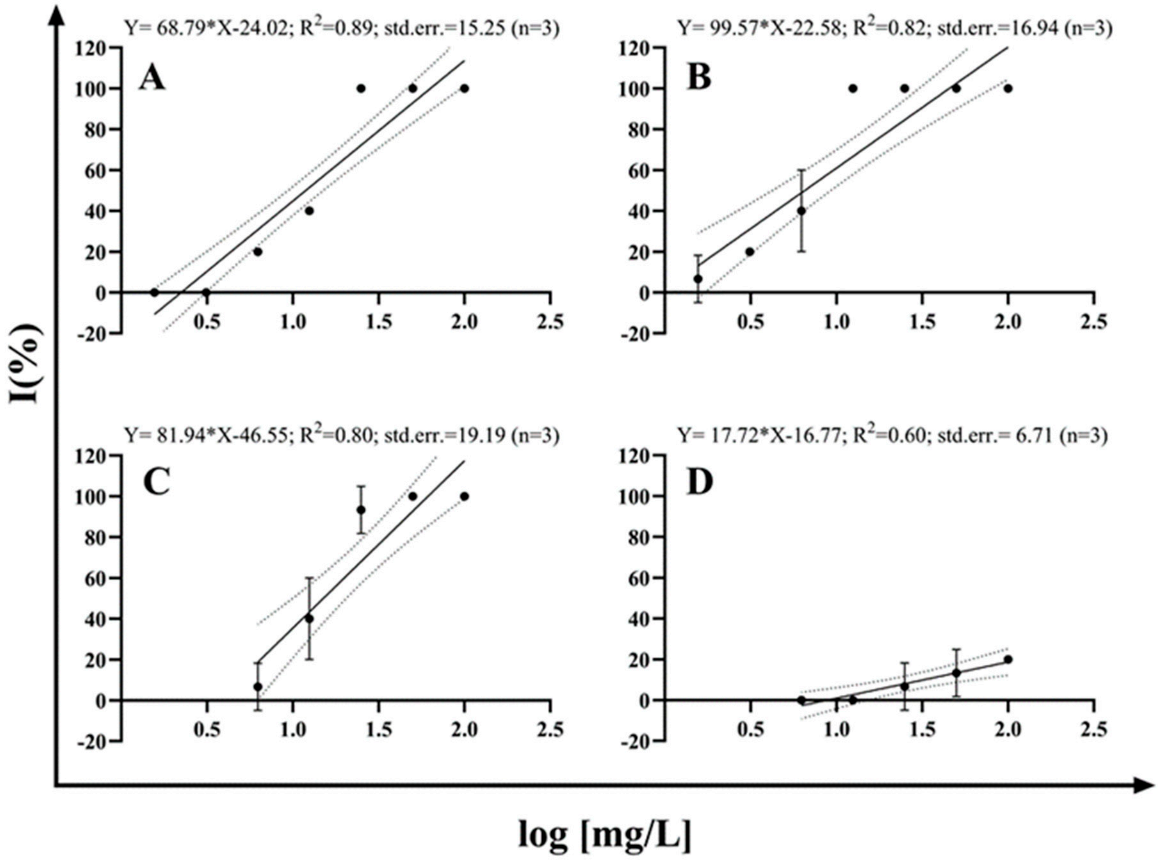

Figure S4. Concentration–response relationship of **1** (A), **2** (B), **3** (C) and **4** (D) exposed to *D. magna*; concentrations in the x-axis are expressed as mg/L: I = immobility. Limits of the 95% confidence intervals are indicated by the dashed line, fitted log-linear model is indicated by the solid line.

The results of the I (%) testing of **1**-**4** on of *D. magna* are reported in Figure S4 (A-D). No significant effects were detected at the first lowest exposure concentrations for **1**-**3**, nor at the other concentrations for **4**. Results with *D. magna* showed that the relative toxicity order was **1**>**2** > **3**> **4**. When daphnids were exposed to **1**, at all exposure concentrations provided evidence of a concentration–response, with significant toxic effect up to 40% at 12.5 mg/L. Indeed. The exposure to **2** showed effects ranged between 6.7% (1.25 mg/L) and 100% (12.5 mg/L). When *D. magna* was exposed to **3**, the effects varied between 6.7% and 100%. For **4**, no significant differences ($p < 0.001$) between concentration were highlighted.

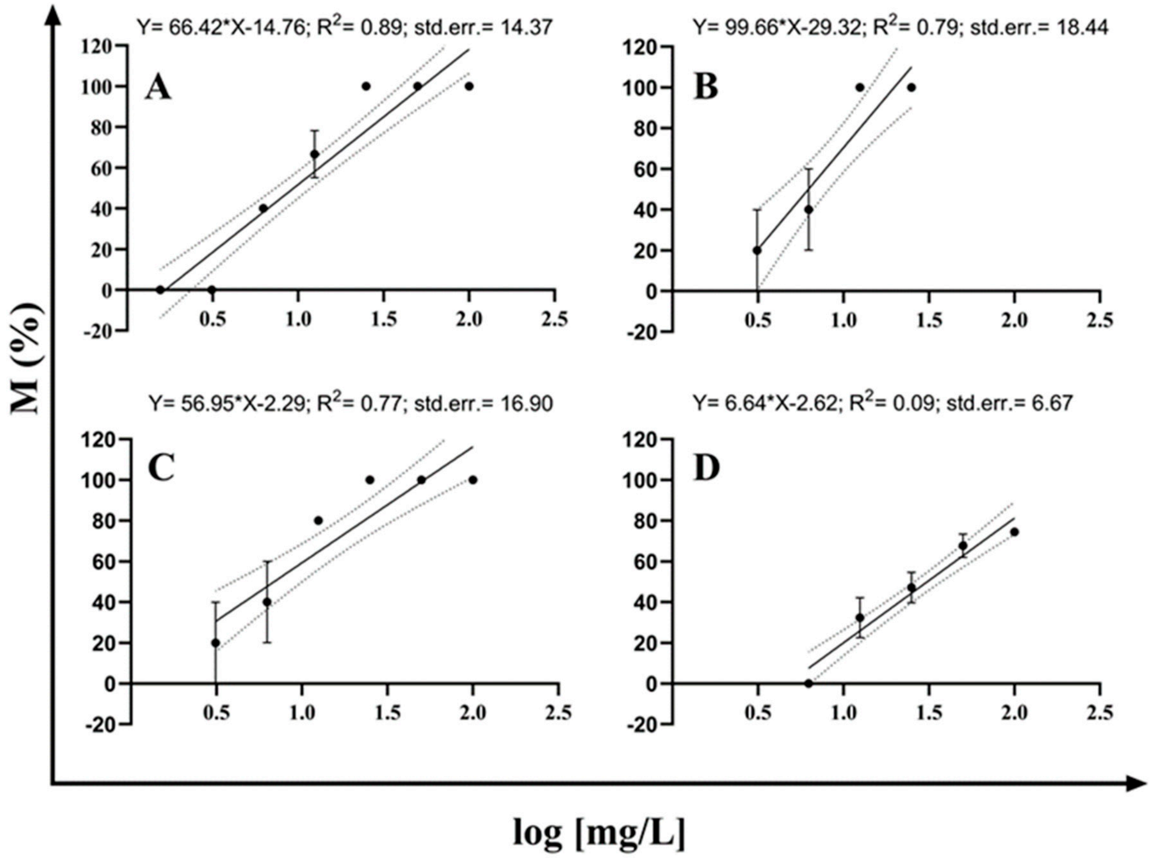

Figure S5. Concentration–response relationship of **1** (A), **2** (B), **3** (C), and **4** (D) exposed to *C. elegans*; concentrations in the x-axis are expressed as mg/L: M = mortality. Limits of the 95% confidence intervals are indicated by the dashed line, fitted log-linear model is indicated by the solid line.

The results about the M (%) of *C. elegans* are reported for **1**-**4** in Figure S5 (A-D). No significant effects were detected at any exposure concentration for **4**. The mortality of nematodes showed a similar toxicity trend for **1** and **3**, but with a higher toxicity level (80%) at 12.5 mg/L for **3**.

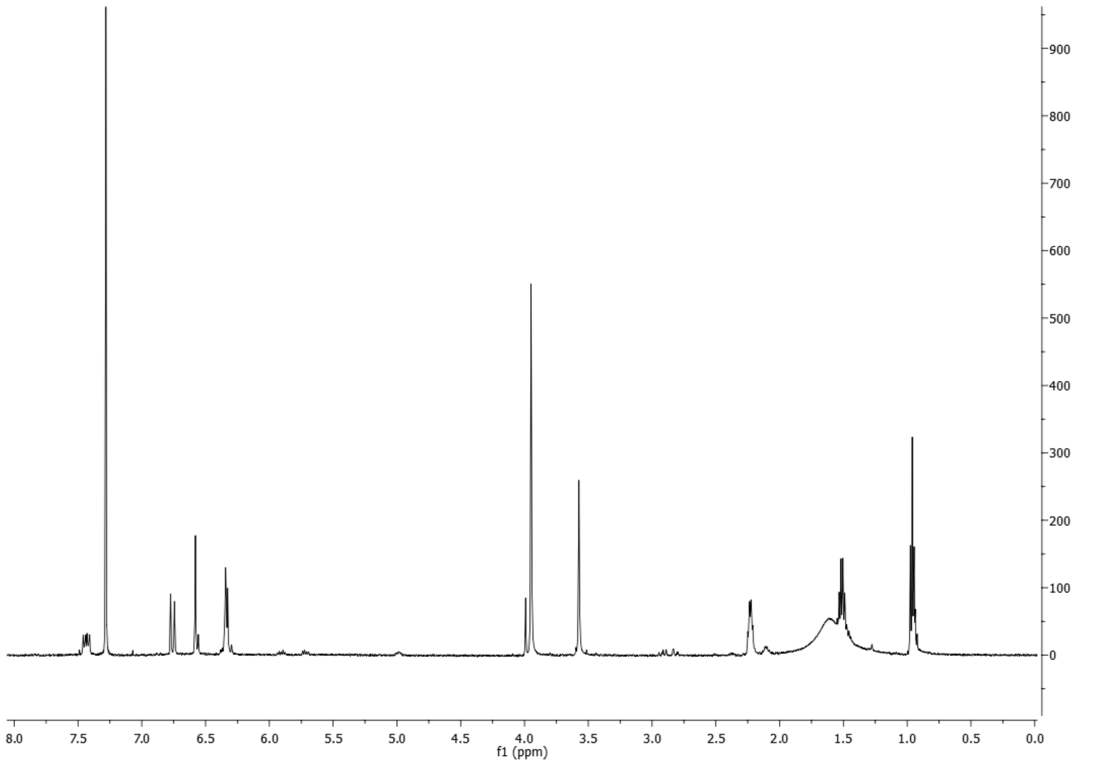

Figure S6. ^{1}H NMR spectrum of cavoxin, **1** ($CDCl_3$, 500 MHz).

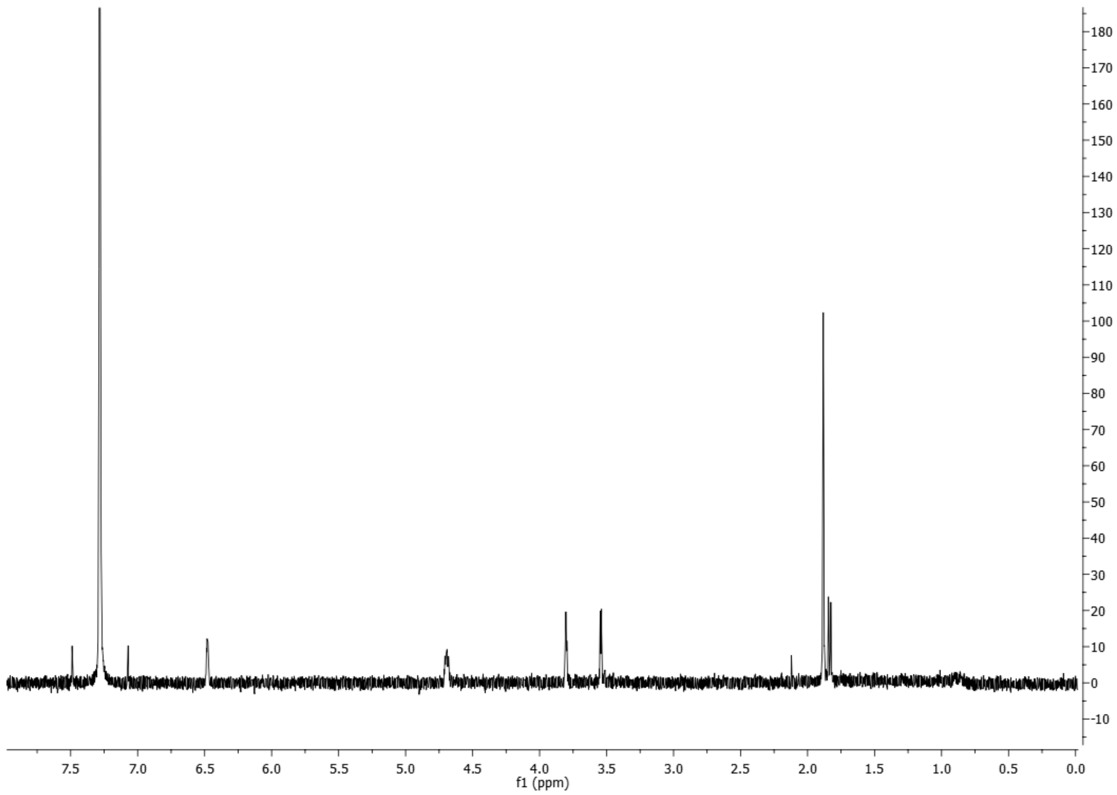

Figure S7. ^{1}H NMR spectrum of *epi*-epoformin, **2** ($CDCl_3$, 500 MHz).

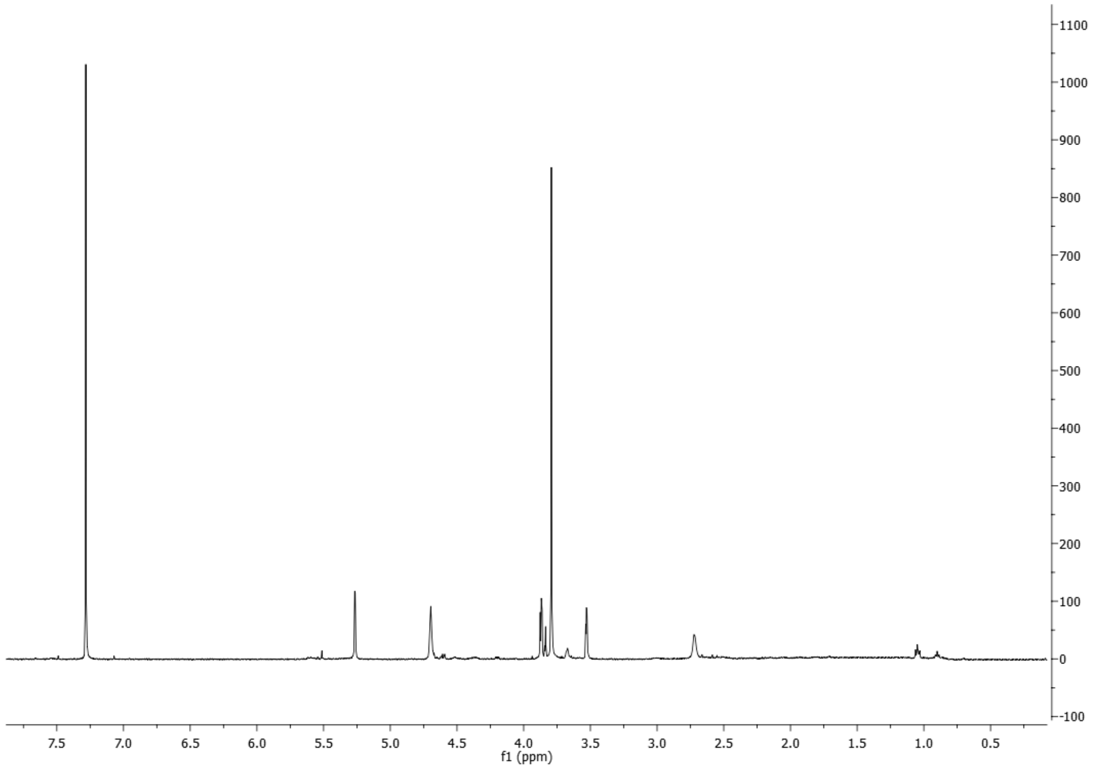

Figure S8. ^{1}H NMR spectrum of sphaeropsidone, **3** ($CDCl_3$, 500 MHz).

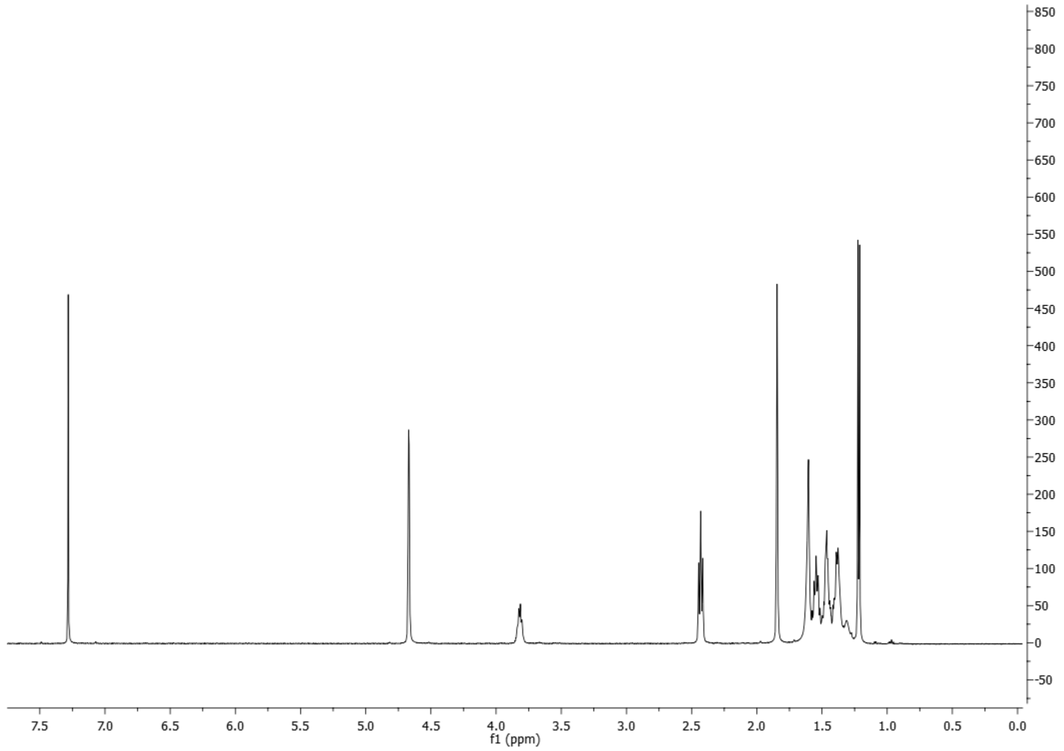

Figure S9. ^{1}H NMR spectrum of seiridin, **4** ($CDCl_3$, 500 MHz).
